# Supplementary material for: Platelet Activation by Antisense Oligonucleotides (ASOs) in the Göttingen Minipig, including an Evaluation of Glycoprotein VI (GPVI) and Platelet Factor 4 (PF4) Ontogeny
Source: Pharmaceutics. 2023 Mar 31;15(4):1112. doi: 10.3390/pharmaceutics15041112 (PMC10143489; doi:10.3390/pharmaceutics15041112)
Supplement: Supplementary file 1 [file pharmaceutics-15-01112-s001.zip › pharmaceutics-2199460-supplementary.pdf]

pharmaceutics

MDPI

Platelet Activation by Antisense Oligonucleotides (ASOs) in the Göttingen Minipig, including an Evaluation of Glycoprotein VI (GPVI) and Platelet Factor 4 (PF4) Ontogeny

Allan Valenzuela, Miriam Ayuso, Laura Buyssens, Chloe Bars, Chris Van Ginneken, Yann Tessier, and Steven Van Cruchten

Table S1. Juvenile Göttingen Minipig sample overview from the in vivo 8-week repeat-dose toxicity study (Valenzuela et al., 2021) [1].

Animal	Age	Sex	Treatment	Platelet count (Giga/L)	GPVI abundance (ng/ml)		PF4 abundance (ng/ml)
					Adjusted to 1 x 10^8 platelets/ml	Adjusted to platelet count in WB	
501	PND 2	male	control	433	h	h	h
511	PND 2	female	control	389	h	h	h
521	PND 2	male	treated	348	h	h	h
531	PND 2	female	treated	344	h	h	h
503	PND 9	male	control	1096	13.14	144.04	2814
513	PND 9	female	control	977	7.81	76.26	3184
523	PND 9	male	treated	969	5.42	52.53	3036
535	PND 9	female	treated	1097	7.93	86.95	2346
504	PND 16	male	control	456	3.38	15.40	2154
517	PND 16	female	control	677	6.80	46.05	1676
524	PND 16	male	treated	546	4.39	23.99	1858
537	PND 16	female	treated	660	14.15	93.42	1182
512	PND 23	female	control	457	24.90	113.79	1017
522	PND 23	male	treated	226	27.32	61.74	1602
532	PND 23	female	treated	581	8.35	48.52	1771
534	PND 23	female	treated	682	50.26	342.77	280
515	PND 29-30	female	control	666	3.44	22.90	2952
527	PND 29-30	male	treated	643	5.51	35.46	790
533	PND 29-30	female	treated	650	37.23	241.97	1392
505	PND 37	male	control	644	5.16	33.23	715
518	PND 37	female	control	517	38.45	198.80	1741
525	PND 37	male	treated	699	9.86	68.91	881
538	PND 37	female	treated	676	15.72	106.23	2313

519	PND 43	female	control	724	23.03	166.75	2300
520	PND 43	female	control	723	4.15	29.98	3113
539	PND 43	female	treated	892	3.86	34.46	3141
514	PND 51	female	control	751	12.84	96.45	1731
516	PND 51	female	control	835	9.94	82.99	2164
526	PND 51	male	treated	828	3.77	31.19	3337
536	PND 51	female	treated	728	3.25	23.69	2987

Abbreviations: WB, whole blood; h, hemolyzed; PND, postnatal day; GPVI, glycoprotein VI; PF4, platelet factor 4.

Reference:

1. Valenzuela, A.; Tardiveau, C.; Ayuso, M.; Buyssens, L.; Bars, C.; van Ginneken, C.; Fant, P.; Leconte, I.; Braendli-Baiocco, A.; Parrott, N.; et al. Safety Testing of an Antisense Oligonucleotide Intended for Pediatric Indications in the Juvenile Göttingen Minipig, Including an Evaluation of the Ontogeny of Key Nucleases. *Pharmaceutics* **2021**, *13*, doi:10.3390/ pharmaceutics13091442.
